# Associations between total dairy, high-fat dairy and low-fat dairy intake, and depressive symptoms: findings from a population-based cross-sectional study

**DOI:** 10.1007/s00394-022-02950-8

**Published:** 2022-08-10

**Authors:** Meghan Hockey, Mohammadreza Mohebbi, Tommi Tolmunen, Sari Hantunen, Tomi-Pekka Tuomainen, Helen Macpherson, Felice N. Jacka, Jyrki K. Virtanen, Tetyana Rocks, Anu Ruusunen

**Affiliations:** 1 IMPACT (The Institute for Mental and Physical Health and Clinical Translation), Food & Mood Centre, Deakin University, Geelong, Australia; 2 Faculty of Health, Biostatistics Unit, Deakin University, Geelong, Australia; 3 Department of Adolescent Psychiatry, Kuopio University Hospital, Kuopio, Finland; 4 Institute of Clinical Medicine / Psychiatry, University of Eastern Finland, Kuopio, Finland; 5 Institute of Public Health and Clinical Nutrition, University of Eastern Finland, Kuopio, Finland; 6 Institute for Physical Activity and Nutrition, School of Exercise and Nutrition Sciences, Deakin University, Geelong, Australia; 7 Murdoch Children’s Research Institute, Centre for Adolescent Health, Melbourne, Australia; 8 Black Dog Institute, Sydney, Australia; 9 Department of Psychiatry, Kuopio University Hospital, Kuopio, Finland

**Keywords:** Dairy, Depression, Dairy fat, Fermented foods

## Abstract

**Purpose:**

Evidence on the association between dairy intake and depression is conflicting. Given numerous dietary guidelines recommend the consumption of low-fat dairy products, this study examined associations between total dairy, high-fat dairy, and low-fat dairy intake and the prevalence of elevated depressive symptoms. Associations between dairy products, which differed in both fat content and fermentation status, and depressive symptoms were also explored.

**Methods:**

This cross-sectional study included 1600 Finnish adults (mean age 63 ± 6 years; 51% female) recruited as part of the Kuopio Ischaemic Heart Disease Risk Factor Study. Dairy intake was assessed using 4-day food records. Elevated depressive symptoms were defined as having a score ≥ 5 on the Diagnostic and Statistical Manual of Mental Disorders-III Depression Scale, and/or regularly using one or more prescription drugs for depressive symptoms.

**Results:**

In total, 166 participants (10.4%) reported having elevated depressive symptoms. Using multivariate logistic regression models, intake in the highest tertile of high-fat dairy products (OR 0.64, 95% CI 0.41–0.998, *p* trend = 0.04) and high-fat non-fermented dairy products (OR 0.60, 95% CI 0.39–0.92, *p* trend = 0.02) were associated with reduced odds for having elevated depressive symptoms. Whereas no significant association was observed between intake of total dairy, low-fat dairy, or other dairy products, and depressive symptoms.

**Conclusion:**

Higher intake of high-fat dairy and high-fat non-fermented dairy products were associated with reduced odds for having elevated depressive symptoms in middle-aged and older Finnish adults. Given the high global consumption of dairy products, and widespread burden of depression, longitudinal studies that seek to corroborate these findings are required.

**Supplementary Information:**

The online version contains supplementary material available at 10.1007/s00394-022-02950-8.

## Introduction

Dairy products are consumed by more than six billion people worldwide [1], and contribute to the dietary intake of protein, fat, and a range of vitamins, minerals and bioactive compounds [2].

Milk fat is a major dietary source of saturated fatty acids (SFAs), therefore, several dietary guidelines around the world recommend the consumption of low-fat dairy products, due to the presumed lowered cardiovascular disease (CVD) risk [3, 4]. However, while recent meta-analyses suggest that whole-fat dairy intake does not adversely affect CVD risk [5], differences between whole- and low-fat dairy intake in relation to other health outcomes is less clear.

Depression is the leading contributor to disease burden globally, and is estimated to impact 300 million people worldwide [6]. While overall diet quality is increasingly recognised as an important modifiable risk factor in the prevention and management of depression [7, 8], the role of commonly consumed foods, such as dairy products, is less clear. A recent systematic review of prospective cohort and cross-sectional studies found most studies reported no significant association between total dairy intake and depression [9]. In contrast, significant associations were observed for milk, yoghurt, and cheese, however, the direction of these associations was not consistent across studies [10–12]. Further, few studies considered the unique nutritional differences between dairy products and examined whether this association differed based on the fat content or fermentation status of dairy products.

To date, only two cross-sectional studies have evaluated associations between whole- and low-fat dairy intake and depressive symptoms [13, 14]. Both studies demonstrated that higher low-fat dairy intake, but not whole-fat dairy intake, was associated with a decreased prevalence of depressive symptoms [13, 14]. However, these studies were conducted in Japanese and Iranian populations who have low habitual dairy intake (per capital milk intake 59 kg/year and 54 kg/year, respectively) relative to populations such as Finland (458 kg/year) [15]. Therefore, it is unclear whether these findings are generalisable to those with higher levels of dairy intake. Further, although fermented dairy products have been shown to favourably influence pathways associated with depression, no studies have considered fermentation status alongside fat content in relation to depressive symptoms. For example, fermented dairy intake has been associated with increased strains of beneficial bacteria within the gut microbiota (e.g., *Lactobacillus* and *Bifidobacterium* [16]) and lower markers of inflammation and oxidative stress [17], which are pathways associated with depression [18, 19]. Therefore, this study aimed to first examine associations between total dairy, high-fat dairy, and low-fat dairy intake and the prevalence of elevated depressive symptoms in middle-aged and older Finnish adults. As a secondary aim, differences between dairy products that also differed in fermentation status were explored.

## Methods

### Study design and population

**Fig. 1:**
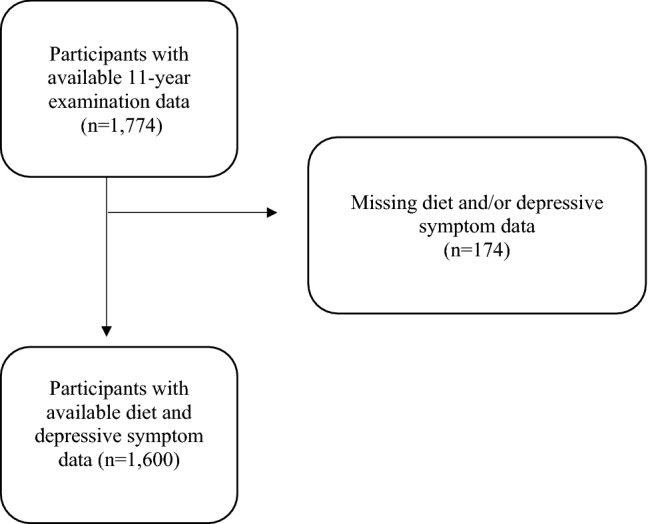
Study flow chart

The Kuopio Ischaemic Heart Disease (KIHD) Risk Factor study is an ongoing population-based cohort study designed to investigate risk factors for CVD and other chronic diseases in middle-aged and older men and women in Finland, and has been previously described elsewhere [20]. Briefly, baseline examinations were conducted between 1984 and 1989 in a sample of men living in Kuopio, Finland, and the neighboring communities. In total, 2682 men (82.9% of those who were eligible to participate in the KIHD study) aged 42, 48, 54 or 60 years at baseline were recruited in two cohorts between 1984–1986 and 1986–1989. Between 1998 and 2001, all men from the second cohort (recruited 1986–1989) were invited to participate in the 11-year re-examinations (*n* = 854, 85.6% participated). Baseline examination data were also collected at this timepoint (1998–2001) for 920 post-menopausal women, aged 53–73 years, from the Kuopio region. The present cross-sectional study was conducted using data obtained between 1998–2001 for men (*n* = 854) and women (*n* = 920). Further longitudinal data were not available for analysis. Participants with missing data on dairy intake or depressive symptoms were excluded (*n* = 174), leaving 1600 participants available for inclusion in this cross-sectional study (see Fig. 1). The KIHD received approval from the research ethics committee of the University of Kuopio and Kuopio University Hospital, and written, informed consent was obtained from all participants. This present study was approved for exemption from ethical review in accordance with the National Statement on Ethical Conduct in Human Research (2007, updated 2018) Section 5.1.22 by the Deakin University Human Research Ethics Committee.

### Dietary assessment

Dietary intakes were assessed using the average of 4-day food records, including 3 weekdays and 1 weekend day. Participants were provided with instructions on how to complete the food records by an experienced nutritionist. The records were cross checked by a nutritionist together with the participant upon completion. NUTRICA® 2.5 (The Social Insurance Institution of Finland, Turku, Finland) was used to quantify food and nutrient composition from the food records. Dairy foods were first categorized based on their fat content as either high-fat (≥ 3.5% fat) or low-fat (< 3.5% fat). The specific foods found within each category are listed in Supplementary Table 1. High-fat and low-fat dairy foods were then combined to produce total dairy intake. We further grouped dairy products according to fat content (high-fat or low-fat) and fermentation status (fermented or non-fermented). Dairy categories were first examined as continuous variables (mean g/day) and then transformed into data-driven tertiles, to aid with clinical interpretation of the data and comparability with the wider literature.

### Depression assessment

Depressive symptoms were assessed using the Diagnostic and Statistical Manual of Mental Disorders, Third Edition (DSM-III), Depression Scale, which is a self-rated questionnaire consisting of 12 symptoms of depression based on the DSM-III diagnostic criteria (range 0–12) [21, 22]. Depressive symptoms were first examined as a continuous variable using the DSM-III Depression Scale. To identify those with elevated depressive symptoms, we combined participants who scored five or higher on the DSM-III and/or regularly used one or more prescription drugs for depressive symptoms. A cut-off score of five or more (scores of 5–12) was used as per prior research in the same population [23].

### Covariate assessment

Important factors in the association between diet and depression were selected a priori, and included age (years), sex (male/female), body mass index (BMI), adulthood socioeconomic status (SES, points), energy intake (kJ/day), fruit, berry, and vegetable intake (g/day), and history of CVD (yes/no). BMI was calculated as the ratio of weight (kg) to the square of height (metres), as measured during KIHD study visits conducted between 1998 and 2001. Adulthood SES was calculated using measures of education, occupation, income, housing tenure, and ownership of material goods [24]. History of CVD was assessed using a self-administered questionnaire with a positive CVD history coded based on the following criteria: (1) at least one clinician-diagnosed CVD condition and (2) used nitrates at least once per week and had angina pectoris according to the World Health Organisation angina pectoris questionnaire (the Rose Angina Questionnaire) [25].

### Statistical analyses

The analysis plan for this study was pre-registered on the Open Science Framework. Continuous non-normal data were expressed as medians (inter-quartile range, IQR) and categorical data as absolute (*n*) and relative frequencies (%). Differences in participant characteristics across tertiles of dairy intake were compared using ANOVA (continuous data) or chi-squared tests (categorical data). Differences in participant characteristics for those with and without elevated depressive symptoms were also compared using non-parametric Mann–Whitney *U* tests and chi-squared tests. Linear regression models were first used to assess the relationship between depressive symptom scores and dairy intake (per 100 g) for each of the different types of dairy products. Estimated standardised regression coefficients (beta co-efficient) and standard errors were used to indicate the association with depressive symptoms score per 100 g unit increase in dairy intake. Odds ratios (OR) and their 95% confidence intervals (CI) were then calculated to evaluate associations between dairy intake, across tertiles of intake, and the prevalence of having elevated depressive symptoms using logistic regression models. The inclusion of covariates within models was informed by the literature and using a Directed Acyclic Graph (DAG) (see Supplementary Fig. 1). While it is acknowledged causality cannot be inferred from cross-sectional data, a DAG was developed to better indicate the relationships providing the best model fit [26]. Model 1 included age, sex, and energy intake. Model 2 included model 1 and further adjustments for BMI, fruit, berry and vegetable intake, adulthood SES, and history of CVD. In all models, the first (lowest) tertile of dairy intake was considered as the reference. Subgroup analysis based on sex were also conducted across tertiles of dairy intake, for the maximally adjusted model (model 2). *P* trends, defined as the *p* value for the test that examines dose–response associations between tertiles and the outcomes, were reported. *P* trend < 0.05 was regarded as statistically significant (two-tailed) and was presented along with 95% CIs. All analyses were performed using SPSS software (version 27).

## Results

This study included 1600 Finnish adults (51% female) with a mean ± SD age of 62.5 ± 6.4 years. In total, 166 participants (10.4%) reported having elevated depressive symptoms, of whom *n* = 121 reported elevated DSM-III scores, *n* = 29 reported using anti-depressants prescribed for depressive symptoms, and *n* = 16 reported both. The median total dairy intake was 448 g/day (IQR 284–631), with low-fat dairy intake more than sevenfold greater that of high-fat dairy intake (median 366 g/day compared to 51 g/day, respectively). Briefly, high-fat milk contributed to the greatest proportion of high-fat dairy intake (66.6%), followed by high-fat cheese (28.9%). Whereas low-fat milk contributed to the greatest proportion of low-fat dairy intake (68.4%).

Table 1 presents the demographic and lifestyle characteristics of participants across tertiles of total dairy, high-fat dairy, and low-fat dairy intake. Participants with higher total dairy intake, were more likely to be male, were older in age, consumed less alcohol, had a higher energy intake and higher SES, when compared to those with lower total dairy intake. Characteristics of all participants, and according to those with and without elevated depressive symptoms, are presented in Supplementary Table 2. Significant differences were observed between groups, with those with elevated depressive symptoms more likely to be female, have a higher SES, have a higher BMI, have a history of CVD, have a lower energy intake, but were less likely to be married or living with their partner, when compared to those without elevated symptoms. High-fat dairy intake was also significantly lower among those with elevated depressive symptoms, however, there was no significant difference in the intake of any other dairy products (Supplementary Table 2).

**Table 1:** Demographic and lifestyle characteristics of KIHD participants (*n* = 1600) presented across tertiles of total dairy, high-fat dairy, and low-fat dairy intake, values presented as median (IQR) or *n* (%)

	Total dairy intake				High-fat dairy intake				Low-fat dairy intake				
	Tertile 1	Tertile 2	Tertile 3	*P* trend	Tertile 1	Tertile 2	Tertile 3	*P* trend	Tertile 1		Tertile 2	Tertile 3	*P* trend
Median (IQR) intake, g/day	212 (141, 284)	448 (393, 500)	723 (631, 885)	–	19 (9, 28)	51 (43, 60)	112 (87, 177)	–	128 (66, 194)		366 (310, 424)	638 (548, 793)	–
Participants, *n*	533	534	533	–	532	535	533	–	533		534	533	–
Sex													
Women	305 (57)	295 (55)	220 (41)	< 0.001^2^	275 (52)	307 (57)	238 (45)	< 0.001^2^	286 (54)		304 (57)	230 (43)	< 0.001^2^
Men	228 (43)	239 (45)	313 (59)		257 (48)	228 (43)	295 (55)		247 (46)		230 (43)	303 (57)	
Age, years	60 (54,66)	61 (55,67)	65 (60, 66)	0.002^1^	65 (60, 67)	61 (55, 66)	60 (54, 66)	0.034^1^	60 (54, 66)		65 (58, 67)	65 (60, 66)	0.002^1^
Energy expenditure for leisure-time physical activity, kcal/d	124 (57, 241)	131 (67, 248)	134 (61, 268)	0.376^1^	124 (61, 242)	127 (66, 249)	141 (57, 275)	0.997^1^	133 (58, 246)		125 (63, 238)	134 (63, 265)	0.214^1^
BMI, kg/m^2^	27 (25, 31)	27 (25, 30)	27 (25, 30)	0.488^1^	28 (25, 31)	27 (25, 30)	27 (24, 30)	0.047^1^	27 (25, 31)		27 (25, 30)	27 (25, 30)	0.896^1^
SES, points	8 (4, 11)	8 (4, 11)	8 (5, 12)	0.020^1^	9 (6, 12)	7 (4, 10)	8 (4, 11)	< 0.001^1^	8 (4, 11)		7 (4, 11)	8 (5, 12)	0.029^1^
Total energy intake, kJ/day	6671 (5269, 8160)	7219 (6080, 8407)	8539 (7321, 10372)	< 0.001^1^	6741 (5413, 8047)	7339 (6134, 8757)	8361 (6967, 9956)	< 0.001^1^	7006 (5640, 8614)		7089 (5877 8407)	8275 (7075, 9991)	< 0.001^1^
Fruit, berry, and vegetable intake^a^, g/day	279 (182, 383)	299 (197, 417)	274 (175, 393)	0.339^1^	256 (165, 365)	315 (215, 425)	274 (176, 395)	< 0.001^1^	275 (169, 382)		305 (203, 417)	274 (183, 392)	0.151^1^
Alcohol intake, g/week	16 (1, 66)	14 (2, 54)	12 (1, 50)	0.029^1^	9 (1, 47)	13 (2, 55)	19 (3, 64)	0.403^1^	16 (2, 67)		14 (2, 56)	11 (1, 47)	0.008^1^
DSM-III depressive symptoms, scores	1 (0, 2)	1 (0, 2)	1 (0, 2)	0.946^1^	1 (0, 2)	1 (0, 2)	1 (0, 2)	0.288^1^	1 (0, 2)		1 (0, 2)	1 (0, 2)	0.822^1^
Anti-depressant medication use^b^	9 (2)	19 (4)	17 (3)	0.148^2^	19 (4)	17 (3)	9 (2)	0.146^2^	10 (2)		19 (4)	16 (3)	0.239^2^
History of CVD	220 (41)	233 (44)	241 (45)	0.426^2^	249 (47)	224 (42)	221 (42)	0.147^2^	218 (41)		243 (46)	233 (44)	0.310^2^
Marital status (married or living as couple)	396 (74)	414 (78)	404 (76)	0.435^2^	394 (74)	413 (77)	407 (76)	0.520^2^	397 (75)		404 (76)	413 (78)	0.409^2^

*BMI* body mass index, *CVD* cardiovascular disease, *DSM-III* depressive symptoms, diagnostic and statistical manual of mental disorders (third edition) depression scale, *KIHD* Kuopio Ischaemic Heart Disease Risk Factor study, *SES* socioeconomic status. % Rounded to nearest whole value and may, therefore, not add exact to 100

^a^Excludes potatoes

^b^Anti-depressant medication use for those using one or more prescription drugs for depressive symptoms only

^1^ANOVA test

^2^ Chi-squared test

Supplementary Table 3 presents the unadjusted beta co-efficient and standard error for depressive symptoms scores for each of the different types of dairy products, examined using linear regression models (results presented as unadjusted values only). In brief, there was no significant linear relationship between depressive symptom scores and any of the dairy variables in the unadjusted models. Table 2 presents the unadjusted and multivariate adjusted logistic regression models for the prevalence of elevated depressive symptoms, across tertiles of dairy intake (ORs, 95% CI). In unadjusted models, there was an inverse relationship between high-fat dairy intake and the prevalence of having elevated depressive symptoms, which was of borderline significance only (OR 0.68, 95% CI 0.45–1.02, *p* trend = 0.05). After adjustment for various demographic and lifestyle factors, this association was slightly changed, with high-fat dairy intake in the highest (median intake 112 g/day) vs lowest tertile (median intake 19 g/day) associated with 36% reduced odds for having elevated depressive symptoms (OR 0.64, 95% CI 0.41–0.998, *p* trend = 0.04, model 2). In contrast, no significant relationship was observed between total dairy or low-fat dairy intake and elevated depressive symptoms, in either unadjusted or adjusted models.

**Table 2:** Unadjusted and multivariate adjusted logistic regression models for the prevalence of elevated depressive symptoms, across tertiles of dairy intake (ORs, 95% CI)

	Intake tertile							
	1	2			3			
	Ref	OR	95% CI	Wald statistic (DF)	OR	95% CI	Wald statistic (DF)	*P *trend
Total dairy intake (g/day)								
Events/participants (*n*)	49/533	56/534			61/533			
Median intake (IQR)	212 (141, 284)	448 (393, 500)			723 (631, 885)			
Mean (SD)	208 (85)	445 (65)			783 (195)			
Unadjusted	1	1.16	0.77–1.73	0.50 (1)	1.28	0.86–1.90	1.45 (1)	0.23
Model 1	1	1.18	0.78–1.78	0.63 (1)	1.47	0.95–2.27	3.05 (1)	0.08
Model 2	1	1.20	0.79–1.83	0.77 (1)	1.42	0.91–2.19	2.46 (1)	0.12
High-fat dairy intake (g/day)								
Events/participants (*n*)	62/532	60/535			44/533			
Median intake (IQR)	19 (9, 28)	51 (43, 60)			112 (87, 177)			
Mean (SD)	18 (11)	51 (10)			174 (160)			
Unadjusted	1	0.96	0.66–1.40	0.05 (1)	0.68	0.45–1.02	3.40 (1)	0.05
Model 1	1	0.83	0.56–1.22	0.91 (1)	0.61	0.39–0.94	5.01 (1)	0.03
Model 2	1	0.91	0.61–1.35	0.22 (1)	0.64	0.41–0.998	3.88 (1)	0.04
Low-fat dairy intake (g/day)								
Events/participants (*n*)	49/533	59/534			58/533			
Median intake (IQR)	128 (66, 194)	366 (310, 424)			638 (548, 793)			
Mean (SD)	130 (77)	368 (64)			694 (188)			
Unadjusted	1	1.23	0.82–1.83	1.01 (1)	1.21	0.81–1.80	0.84 (1)	0.38
Model 1	1	1.21	0.80–1.81	0.81 (1)	1.31	0.86–2.00	1.59 (1)	0.21
Model 2	1	1.22	0.81–1.85	0.94 (1)	1.27	0.83–1.94	1.17 (1)	0.27
High-fat fermented dairy intake								
Events/participants (*n*)	60/533	53/533			53/534			
Median intake (IQR)	3 (0, 8)	21 (16, 26)			48 (38, 65)			
Mean (SD)	4 (4)	21 (6)			57 (30)			
Unadjusted	1	0.87	0.59–1.29	0.48 (1)	0.87	0.59–1.28	0.50 (1)	0.51
Model 1	1	0.69	0.46–1.03	3.24 (1)	0.71	0.47–1.08	2.57 (1)	0.15
Model 2	1	0.78	0.51–1.17	1.49 (1)	0.84	0.55–1.28	0.68 (1)	0.48
Low-fat fermented dairy intake								
Events/participants (*n*)	64/534	44/517			58/549			
Median intake (IQR)	0 (0, 0)	75 (50, 100)			259 (194, 362)			
Mean (SD)	0 (2)	76 (35)			296 (143)			
Unadjusted	1	0.68	0.46–1.02	3.41 (1)	0.87	0.60–1.27	0.55 (1)	0.74
Model 1	1	0.52	0.34–0.79	9.23 (1)	0.72	0.49–1.06	2.71 (1)	0.37
Model 2	1	0.55	0.36–0.84	7.64 (1)	0.76	0.52–1.13	1.82 (1)	0.50
High-fat non-fermented dairy intake								
Events/participants (*n*)	67/534	55/533			44/533			
Median intake (IQR)	2 (0, 6)	21 (16, 27)			69 (47, 136)			
Mean (SD)	3 (3)	22 (7)			137 (168)			
Unadjusted	1	0.80	0.55–1.17	1.30 (1)	0.63	0.42–0.94	5.20 (1)	0.03
Model 1	1	0.72	0.49–1.05	2.88 (1)	0.60	0.39–0.91	5.88 (1)	0.03
Model 2	1	0.78	0.52–1.15	1.58 (1)	0.60	0.39–0.92	5.56 (1)	0.02
Low-fat non-fermented dairy intake								
Events/participants (*n*)	48/533	56/534			62/533			
Median intake (IQR)	55 (28, 85)	206 (155, 265)			493 (395, 637)			
Mean (SD)	56 (33)	211 (60)			547 (199)			
Unadjusted	1	1.18	0.79–1.78	0.66 (1)	1.33	0.89–1.98	1.98 (1)	0.17
Model 1	1	1.14	0.76–1.73	0.40 (1)	1.50	0.99–2.27	3.64 (1)	0.049
Model 2	1	1.12	0.74–1.69	0.26 (1)	1.44	0.95–2.18	2.90 (1)	0.08

Total N for all models was 1600. Model 1 adjusted for age, sex, and energy intake. Model 2 adjusted for model 1 and BMI, fruit, berry and vegetable intake, SES, and history of CVD

When the fermentation status of dairy products was also considered, high-fat non-fermented dairy intake was inversely associated with elevated depressive symptoms in the unadjusted model (OR 0.63, 95% CI 0.42–0.94, *p* trend = 0.03). In the final model, this association was not appreciably changed, with high-fat non-fermented dairy intake in the highest (median intake 69 g/day) vs lowest tertile (median intake 2g/day) associated with 40% reduced odds for having elevated depressive symptoms (OR 0.60, 95% CI 0.39–0.92, *p* trend = 0.02, model 2). Whereas we observed no significant association between low-fat fermented dairy, high-fat fermented dairy, or low-fat non-fermented dairy intake, and the prevalence of having elevated depressive symptoms. After further adjustments for alcohol intake, low-fat non-fermented dairy intake was associated with increased odds for having elevated depressive symptoms (OR 1.57, 95% CI 1.03–2.41), although estimates for other dairy products were not significantly changed. We also tested associations by including examination year in Model 1 adjustments, however, this did not markedly change estimates (data not shown).

### Subgroup analyses

When groups were stratified by sex, high-fat fermented dairy intake in the second tertile in men, and low-fat fermented dairy intake in the second tertile in women, were associated with lower odds for depression (see Supplementary Table 4)*.* However, the overall test for these relationships were not statistically significant (*p trend*= 0.16 and *p trend* > 0.99, respectively). Similarly, the ORs for other dairy products were not statistically significant.

## Discussion

In this population-based cross-sectional study of middle-aged and older Finnish adults, higher high-fat dairy intake, but not total dairy or low-fat dairy intake, was associated with reduced odds for having elevated depressive symptoms. This association remained statistically significant after adjustment for age, sex, energy intake, BMI, fruit, berry and vegetable intake, SES, and history of CVD. When the fermentation status of dairy products was also considered, higher high-fat non-fermented dairy intake was associated with reduced odds for having elevated depressive symptoms. Low-fat non-fermented dairy intake was associated with increased odds for having elevated depressive symptoms after further adjustments for alcohol intake only. To our knowledge, this study is the first to examine the association between dairy products that differed in fat content (high fat vs low fat) and fermentation status (fermented vs non-fermented) with depressive symptoms, in a population with a known high level of dairy intake.

Several dietary guidelines recommend the consumption of low-fat dairy products in place of whole-fat equivalents [3, 4]. In juxtaposition with these guidelines, the present study found that high-fat dairy intake, but not low-fat dairy intake, was inversely associated with having elevated depressive symptoms. In contrast to our findings, a previous cross-sectional study in Japanese adults (*n* = 1159) observed that low-fat dairy intake in the highest tertile (≥ 4 times/week) was associated with a lower prevalence of depressive symptoms, when compared to no intake [13]. Whereas no significant association was observed between whole-fat dairy intake (≥ 4 times/week) and the prevalence of depressive symptoms [13]. A further cross-sectional study in Iranian military personnel (*n* = 230) similarly reported that higher low-fat dairy intake (mean 338 g/day), but not whole-fat dairy intake, was associated with reduced odds for depressive symptoms [14]. While it is possible our findings are due to chance, potential explanations for these discrepancies could include our larger sample size, differences in dietary assessment tools, or differences in dairy consumption levels between studies. Although inconsistencies in measures used to report dairy intake limit comparability between all studies (e.g., grams per day vs times per week), low-fat dairy intake in the present study (mean ± SD intake of 694 ± 188 g/day in the highest tertile) was more than double that of other studies [14]. It is also plausible that these discordant findings could be explained by differences in the categorization of high- and low-fat dairy products, and the proportion of individual dairy products that contribute to overall intake. For example, one cross-sectional study included whole-fat milk and yoghurt in the classification of high-fat dairy products [13], whereas the present study included a breadth of high-fat dairy products, such as high-fat milk, yoghurt, cheese, and buttermilk (see Supplementary Table 1 for further details). However, no association was found between high-fat dairy intake and depressive symptoms using linear regression models in this study. Therefore, further confirmatory studies are required to replicate these findings.

We also observed an inverse association between high-fat non-fermented dairy intake, but not high-fat fermented dairy intake, and the prevalence of elevated depressive symptoms. Fermented dairy products contain several properties that may confer benefits for depression [27], including living microorganisms with potential probiotic effects, substances that enhance the growth of beneficial bacteria within the gastrointestinal tract (prebiotics), and bioactive metabolites (biogenics) including certain vitamins, bioactive peptides, organic acids, or fatty acids that arise during fermentation [28, 29]. Thus further adequately powered studies are required to investigate potential differences between fermented and non-fermented dairy intake in relation to depression.

Several factors may explain the inverse association observed between high-fat dairy, and high-fat non-fermented dairy intake, and depressive symptoms. First, it is possible these results are a function of reverse causality whereby individuals without chronic health conditions, including depression, may not be engaged within the health service and receive dietary advice to limit the consumption of high-fat dairy products. It is also possible that those with, or at risk of, depression may have altered their diets to be more healthful, and in turn, consumed low-fat dairy products in place of high-fat equivalents. This is concordant with health behaviours among individuals with depression in previous research [30]. Second, given high-fat dairy intake has recently been linked to a lower prevalence of metabolic syndrome, hypertension, and diabetes, it is possible that increased consumption of high-fat dairy products may reduce the prevalence of depressive symptoms via potential indirect benefits to metabolic health [31]. Third, dairy fat is complex and while long-chain saturated fats (SFAs) have been associated with increased CVD risk, the potentially deleterious effects of these SFAs in dairy products may be blunted by the synergistic effects of other fatty acids and bioactive compounds within the high-fat dairy matrix [32]. Compared to long-chain SFAs, short-, medium-, and branched-chain SFAs, and natural trans fatty acids, have different metabolic and physiological effects, that may confer benefits for depression [33, 34]. For example, although further research in humans is required, components naturally present within milk fat such as butyric acid (C4:0), milk fat globule membrane, and dietary *cis-9, trans-11* conjugated linoleic acid, are thought to have anti-inflammatory properties, which is a central pathway in the pathogenesis of depression [18, 35–37].

This study has several important strengths. Our study is the first to examine the association between dairy products, which differed in both fat content and fermentation status, and the prevalence of elevated depressive symptoms. Four-day food records were used to assess dietary intake, which measures actual food intake, and is, therefore, less prone to memory or recall bias. Using this method also allowed us to capture detailed dietary data on the broad range of dairy foods consumed within the Finnish diet. We also adjusted for several lifestyle and demographic factors in our analyses, including fruit, berry, and vegetable intake, which was used as a proxy for overall diet quality.

However, the following limitations should be considered alongside the interpretation of our findings. First, due to the cross-sectional nature of this study, reverse causality cannot be excluded and the direction of the association between dairy intake and depression cannot be ascertained. Therefore, adequately powered prospective studies are required to corroborate these findings. Second, residual confounding cannot be excluded, and it is plausible other components within the dairy matrix (e.g., probiotics, sugar content), or other unknown factors not adjusted for, may have influenced the relationship between dairy intake and depressive symptoms. Further, our finding of an inverse association between high-fat dairy intake and elevated depressive symptoms should be interpreted with caution, given that high-fat dairy intake in the highest tertile was relatively low (mean intake 174 g/day), in comparison to population guidelines for the consumption of dairy intake (e.g., Finnish dietary guidelines recommend 500–600 ml of milk and 2–3 slices of cheese per day) [38]. Lastly, findings may not be generalisable to younger adults and other population groups, particularly given that the types of dairy products consumed can vary considerably across regions.

In this population-based cross-sectional study in middle-aged and older Finnish adults, higher high-fat dairy intake, and high-fat non-fermented dairy intake, were associated with reduced odds for having elevated depressive symptoms. No statistically significant relationship was observed between total, low-fat, low-fat fermented, high-fat non-fermented, or low-fat non-fermented dairy intake and the prevalence of elevated depressive symptoms. Although numerous dietary guidelines recommend the consumption of low-fat dairy products, these findings suggest that high-fat dairy products may confer benefits for depressive symptoms. Further, this research provides preliminary data suggesting that the fat content and fermentation status of dairy products may be important in influencing the association between dairy intake and depression. Given the high global consumption of dairy products, and widespread burden of depression, future prospective studies that seek to clarify the role of dairy intake in relation to depression risk are of high public health interest.

## Supplementary Information

Below is the link to the electronic supplementary material.

Supplementary file1 (DOCX 750 KB)

## Data Availability

Data described in this manuscript will not be made available because it contains sensitive personal data of the participants, which cannot be completely anonymized.
