# Supplementary material for: Associations between total dairy, high-fat dairy and low-fat dairy intake, and depressive symptoms: findings from a population-based cross-sectional study
Source: Eur J Nutr. 2022 Aug 10;62(1):227–37. doi: 10.1007/s00394-022-02950-8 (PMC9899713; doi:10.1007/s00394-022-02950-8)
Supplement: Supplementary file 1 — Supplementary file1 (DOCX 750 KB) [file 394_2022_2950_MOESM1_ESM.docx]

**Supplementary Material**

Associations between total dairy, high-fat dairy and low-fat dairy intake and depressive symptoms: Findings from a population-based cross-sectional study

**Hockey et al.**

**Supplementary Table 1**. Food group classification of dairy products

**Supplementary Table 2.** Demographic and lifestyle characteristics of all KIHD participants (n=1600) and presented for those with (n=166) and without (n=1434) elevated depressive symptoms, median (IQR) or n (%)

**Supplementary Table 3.** Unadjusted beta co-efficient and standard error for depressive symptoms score across different types of dairy intake (per 100g)

**Supplementary Table 4**. Multivariate adjusted logistic regression models for the prevalence of elevated depressive symptoms across tertiles of dairy intake, presented according to sex (ORs, 95% CI)

**Supplementary Table 5**. STROBE-nut: An extension of the STROBE statement for nutritional epidemiology

**Supplementary Figure 1:** Directed Acyclic Graph

**Supplementary Table 1**. Food group classification of dairy products

| Dairy category | Dairy subcategory | Dairy products |
| --- | --- | --- |
| High-fat dairy* | | |
|  | *Fermented dairy* | Cheese † |
|  |  | Crème fraiche and sour cream |
|  |  | High-fat cultured buttermilk |
|  |  | High-fat yoghurt |
|  |  |  |
|  | *Non-fermented dairy* | High-fat milk^ |
|  |  | Ice-cream |
|  |  | Cream |
|  |  | Colostrum |
|  |  | Bread cheese/Finnish squeaky cheese |
|  |  |  |
| Low-fat dairy** |  |  |
|  | *Fermented dairy* | Sour milk (≤2.5% fat) |
|  |  | Low-fat cultured buttermilk (≤2.5% fat) |
|  |  | Low-fat and fat-free yoghurt (≤2.5% fat) |
|  |  | Kefir |
|  |  | Curd cheese/quark |
|  |  | Cottage cheese |
|  |  |  |
|  | *Non-fermented dairy* | Low-fat and fat-free milk^#^ (≤1.9% fat) |
|  |  | Powdered milk and milk-based convenience food soups |
|  |  |  |

**High-fat dairy products (≥3.5 % fat); **Low-fat dairy products (<3.5% fat); †Includes all fermented cheese products like Blue, Edam, Gouda and Swiss cheese; ^Includes farm milk (unpasteurized whole milk), whole milk and cream milk; ^#^Includes semi-skimmed milk and skimmed milk.*

**Supplementary Table 2.** Demographic and lifestyle characteristics of all KIHD participants (n=1600) and presented for those with (n=166) and without (n=1434) elevated depressive symptoms, median (IQR) or n (%)

| Characteristics | All  *(n=1600)* | Without elevated depressive symptoms  *(n=1434)* | With elevated depressive symptoms *(n=166)* | P-value |
| --- | --- | --- | --- | --- |
| Age, years | 55 (61, 66) | 61 (59, 66) | 61 (54, 66) | 0.321^1^ |
| Sex  Women, n (%)  Men, n (%) | 820 (51)  780 (49) | 698 (49)  736 (51) | 122 (74)  44 (27) | <0.001^2^ |
| Total energy intake, kJ/day | 7375  (6048, 9006) | 7416  (6082, 9052) | 7119  (5803, 8418) | 0.024^1^ |
| Total dairy intake, g/day | 448 (284, 631) | 447 (281, 632) | 462 (310, 628) | 0.716^1^ |
| High-fat dairy intake, g/day | 51 (28, 87) | 51 (28, 89) | 45 (24, 77) | 0.047^1^ |
| Low-fat dairy intake, g/day | 366 (194, 549) | 363 (189, 547) | 382 (231, 560) | 0.392^1^ |
| High-fat fermented dairy intake, g/day | 21 (8, 38) | 21 (8, 38) | 19 (8, 37) | 0.435^1^ |
| Low-fat fermented dairy intake, g/day | 75 (0, 200) | 75 (0, 200) | 68 (0, 196) | 0.503^1^ |
| High-fat non-fermented dairy intake, g/day | 21 (6, 47) | 21 (6, 47) | 18 (5, 37) | 0.081^1^ |
| Low-fat non-fermented dairy intake, g/day | 206 (85, 395) | 203 (82, 394) | 239 (97, 432) | 0.237^1^ |
| Fruit, berry, and vegetable intake^^^, g/day | 278 (183, 399) | 278 (181, 399) | 273 (189, 395) | 0.703^1^ |
| Alcohol intake, g/week | 13 (1, 56) | 14 (2, 56) | 10 (0, 57) | 0.295^1^ |
| BMI, kg/m^2^ | 27 (25, 30) | 27 (25, 30) | 28 (25, 32) | 0.013^1^ |
| Energy expenditure* (kcal/d) | 129 (60, 252) | 129 (59, 250) | 135 (62, 286) | 0.519^1^ |
| Smoking status (% smoker) | 185 (12) | 164 (12) | 21 (13) | 0.607^2^ |
| Married or living with partner | 1214 (76) | 1103 (77) | 111 (67) | 0.001^2^ |
| SES, points | 8 (5, 11) | 8 (4, 11) | 9 (5, 13) | 0.004^1^ |
| DSM-III depressive symptom scores | 1 (0,2) | 1 (0, 2) | 6 (5,7) | <0.001^1^ |
| Anti-depressant medication use^#^, n (%) | 45 (2.8) | 0 (0) | 45 (27) | <0.001^2^ |
| History of CVD, n (%) | 694 (43) | 603 (42) | 91 (55) | 0.002^2^ |

BMI, body mass index; CVD, cardiovascular disease; DSM-III scores, Diagnostic and Statistical Manual of Mental Disorders (Third Edition) Depression Scale; KIHD, Kuopio Ischaemic Heart Disease Risk Factor Study; SES, socioeconomic status; *Energy expenditure for leisure-time physical activity; ^^^Excludes potatoes; ^#^Anti-depressant medication use for those using one or more prescription drugs for depressive symptoms only

^1^Mann-Whitney U test; ^2^Chi squared test; % Rounded to nearest whole value and may therefore not add exact to 100

**Supplementary Table 3.** Unadjusted beta co-efficient and standard error for depressive symptoms score across different types of dairy intake (per 100g)

| Dairy category | Beta co-efficient | SE | P value | T statistic | Partial eta squared |
| --- | --- | --- | --- | --- | --- |
| Total dairy intake | -0.008 | 0.018 | 0.642 | -0.466 | 0.000 |
| High-fat dairy intake | -0.037 | 0.041 | 0.376 | -0.886 | 0.000 |
| Low-fat dairy intake | -0.002 | 0.018 | 0.928 | -0.090 | 0.000 |
| High-fat fermented dairy intake | -0.216 | 0.165 | 0.190 | -1.310 | 0.001 |
| Low-fat fermented dairy intake | -0.024 | 0.031 | 0.437 | -0.777 | 0.000 |
| High-fat non-fermented dairy intake | -0.024 | 0.042 | 0.572 | -0.565 | 0.000 |
| Low-fat non-fermented dairy intake | 0.008 | 0.020 | 0.689 | 0.400 | 0.000 |

**Supplementary Table 4.** Multivariate adjusted logistic regression models for the prevalence of elevated depressive symptoms across tertiles of dairy intake, presented according to sex (n=780 men, n=820 women) (ORs, 95% CI) *

|  | 1 | 2 | | 3 | |  |
| --- | --- | --- | --- | --- | --- | --- |
|  | **Ref** | **OR** | **95% CI** | **OR** | **95% CI** | **p-trend** |
| Men | | | | | | |
| Total dairy intake | 1 | 1.64 | 0.72-3.75 | 1.48 | 0.63-3.46 | 0.42 |
| High-fat dairy intake | 1 | 0.78 | 0.35-1.76 | 0.72 | 0.34-1.53 | 0.42 |
| Low-fat dairy intake | 1 | 1.50 | 0.68-3.34 | 1.23 | 0.58-2.84 | 0.57 |
| High-fat fermented dairy intake | 1 | 0.36 | 0.14-0.91 | 0.57 | 0.26-1.21 | 0.16 |
| Low-fat fermented dairy intake | 1 | 0.69 | 0.31-1.55 | 0.63 | 0.30-1.31 | 0.24 |
| High-fat non-fermented dairy intake | 1 | 0.94 | 0.45-1.97 | 0.59 | 0.27-1.29 | 0.17 |
| Low-fat non-fermented dairy intake | 1 | 1.03 | 0.44-2.42 | 1.66 | 0.79-3.52 | 0.14 |
| Women | | | | | | |
| Total dairy intake | 1 | 1.04 | 0.64-1.69 | 1.46 | 0.87-2.44 | 0.15 |
| High-fat dairy intake | 1 | 0.95 | 0.60-1.51 | 0.61 | 0.35-1.05 | 0.06 |
| Low-fat dairy intake | 1 | 1.12 | 0.70-1.81 | 1.30 | 0.78-2.15 | 0.32 |
| High-fat fermented dairy intake | 1 | 1.02 | 0.63-1.66 | 1.04 | 0.62-1.76 | 0.88 |
| Low-fat fermented dairy intake | 1 | 0.54 | 0.33-0.88 | 0.83 | 0.52-1.33 | 1.00 |
| High-fat non-fermented dairy intake | 1 | 0.72 | 0.45-1.15 | 0.61 | 0.36-1.02 | 0.08 |
| Low-fat non-fermented dairy intake | 1 | 1.10 | 0.68-1.78 | 1.34 | 0.81-2.22 | 0.25 |

*****Results presented for model 2, adjusted for age, sex, energy intake, BMI, fruit, berry and vegetable intake, SES, and history of CVD.

**Supplementary Table 5.** STROBE-nut: An extension of the STROBE statement for nutritional epidemiology

Lachat C et al. (2016) STrengthening the Reporting of OBservational studies in Epidemiology – Nutritional Epidemiology (STROBE-nut): an extension of the STROBE statement. Plos Medicine 13(6) <http://dx.doi.org/10.1371/journal.pmed.1002036> [pdf](http://journals.plos.org/plosmedicine/article/asset?id=10.1371%2Fjournal.pmed.1002036.PDF) or [online](http://journals.plos.org/plosmedicine/article?id=10.1371/journal.pmed.1002036) version.

| **Item** | **Item nr** | **STROBE recommendations** | **Extension for Nutritional Epidemiology studies (STROBE-nut)** | **Reported on page #** |
| --- | --- | --- | --- | --- |
| **Title and**  **abstract** | 1 | (a) Indicate the study’s design with a commonly used term in the title or the abstract.  (b) Provide in the abstract an informative and balanced summary of what was done and what was found. | **nut-1** State the dietary/nutritional assessment method(s) used in the title, abstract, or keywords. | 1-2 |
| **Introduction** |  |  |  |  |
| Background rationale | 2 | Explain the scientific background and rationale for the investigation being reported. |  | 4-5 |
| Objectives | 3 | State specific objectives, including any pre-specified hypotheses. |  | 5 |
| **Methods** |  |  |  |  |
| Study design | 4 | Present key elements of study design early in the paper. |  | 5-6 |
| Settings | 5 | Describe the setting, locations, and relevant dates, including periods of recruitment, exposure, follow-up, and data collection. | **nut-5** Describe any characteristics of the study settings that might affect the dietary intake or nutritional status of the participants, if applicable. | 5-6 |
| Participants | 6 | a) Cohort study—Give the eligibility criteria, and the sources and methods of selection of participants. Describe methods of follow-up.  Case-control study—Give the eligibility criteria, and the sources and methods of case ascertainment and control selection. Give the rationale for the choice of cases and controls.  Cross-sectional study—Give the eligibility criteria, and the sources and methods of selection of participants.  (b) Cohort study—For matched studies, give matching criteria and number of exposed and unexposed.  Case-control study—For matched studies, give matching criteria and the number of controls per case. | **nut-6** Report particular dietary, physiological or nutritional characteristics that were considered when selecting the target population. | 5-6 |
| Variables | 7 | Clearly define all outcomes, exposures, predictors, potential confounders, and effect modifiers. Give diagnostic criteria, if applicable. | **nut-7.1** Clearly define foods, food groups, nutrients, or other food components.  **nut-7.2** When using dietary patterns or indices, describe the methods to obtain them and their nutritional properties. | 6-7, Supplementary Table 1 |
| Data sources - measurements | 8 | For each variable of interest, give sources of data and details of methods of assessment (measurement).Describe comparability of assessment methods if there is more than one group. | **nut-8.1** Describe the dietary assessment method(s), e.g., portion size estimation, number of days and items recorded, how it was developed and administered, and how quality was assured. Report if and how supplement intake was assessed.  **nut-8.2** Describe and justify food composition data used. Explain the procedure to match food composition with consumption data. Describe the use of conversion factors, if applicable.  **nut-8.3** Describe the nutrient requirements, recommendations, or dietary guidelines and the evaluation approach used to compare intake with the dietary reference values, if applicable.  **nut-8.4** When using nutritional biomarkers, additionally use the STROBE Extension for Molecular Epidemiology (STROBE-ME). Report the type of biomarkers used and their usefulness as dietary exposure markers.  **nut-8.5** Describe the assessment of nondietary data (e.g., nutritional status and influencing factors) and timing of the assessment of these variables in relation to dietary assessment.  **nut-8.6** Report on the validity of the dietary or nutritional assessment methods and any internal or external validation used in the study, if applicable. | 6-8 |
| Bias | 9 | Describe any efforts to address potential sources of bias. | **nut-9** Report how bias in dietary or nutritional assessment was addressed, e.g., misreporting, changes in habits as a result of being measured, or data imputation from other sources | 7 |
| Study Size | 10 | Explain how the study size was arrived at. |  | 6, Figure 1 |
| Quantitative variables | 11 | Explain how quantitative variables were handled in the analyses. If applicable, describe which groupings were chosen and why. | **nut-11** Explain categorization of dietary/nutritional data (e.g., use of N-tiles and handling of nonconsumers) and the choice of reference category, if applicable. | 6-7 |
| Statistical  Methods | 12 | (a) Describe all statistical methods, including those used to control for confounding  (b) Describe any methods used to examine subgroups and interactions.  (c) Explain how missing data were addressed.  (d) Cohort study—If applicable, explain how loss to follow-up was addressed.  Case-control study—If applicable, explain how matching of cases and controls was addressed.  Cross-sectional study—If applicable, describe analytical methods taking account of sampling strategy.  (e) Describe any sensitivity analyses. | **nut-12.1** Describe any statistical method used to combine dietary or nutritional data, if applicable.  **nut-12.2** Describe and justify the method for energy adjustments, intake modeling, and use of weighting factors, if applicable.  **nut-12.3** Report any adjustments for measurement error, i.e,. from a validity or calibration study. | 8-9 |
| **Results** |  |  |  |  |
| Participants | 13 | (a) Report the numbers of individuals at each stage of the study—e.g., numbers potentially eligible, examined for eligibility, confirmed eligible, included in the study, completing follow-up, and analyzed.  (b) Give reasons for non-participation at each stage.  (c) Consider use of a flow diagram. | **nut-13** Report the number of individuals excluded based on missing, incomplete or implausible dietary/nutritional data. | 6, Figure 1 |
| Descriptive data | 14 | (a) Give characteristics of study participants (e.g., demographic, clinical, social) and information on exposures and potential confounders  (b) Indicate the number of participants with missing data for each variable of interest  (c) Cohort study—Summarize follow-up time (e.g., average and total amount) | **nut-14** Give the distribution of participant characteristics across the exposure variables if applicable. Specify if food consumption of total population or consumers only were used to obtain results. | 9-10 |
| Outcome data | 15 | Cohort study—Report numbers of outcome events or summary measures over time.  Case-control study—Report numbers in each exposure category, or summary measures of exposure.  Cross-sectional study—Report numbers of outcome events or summary measures. |  | 9 |
| Main results | 16 | (a) Give unadjusted estimates and, if applicable, confounder-adjusted estimates and their precision (e.g., 95% confidence interval).  Make clear which confounders were adjusted for and why they were included.  (b) Report category boundaries when continuous variables were categorized.  (c) If relevant, consider translating estimates of relative risk into absolute risk for a meaningful time period. | **nut-16** Specify if nutrient intakes are reported with or without inclusion of dietary supplement intake, if applicable. | 13 |
| Other analyses | 17 | Report other analyses done—e.g., analyses of subgroups and interactions and sensitivity analyses. | **nut-17** Report any sensitivity analysis (e.g., exclusion of misreporters or outliers) and data imputation, if applicable. | 14 (subgroup) |
| **Discussion** |  |  |  |  |
| Key results | 18 | Summarize key results with reference to study objectives. |  | 17 |
| Limitation | 19 | Discuss limitations of the study, taking into account sources of potential bias or imprecision. Discuss both direction and magnitude of any potential bias. | **nut-19** Describe the main limitations of the data sources and assessment methods used and implications for the interpretation of the findings. | 20 |
| Interpretation | 20 | Give a cautious overall interpretation of results considering objectives, limitations, multiplicity of analyses, results from similar studies, and other relevant evidence. | **nut-20** Report the nutritional relevance of the findings, given the complexity of diet or nutrition as an exposure. | 20-21 |
| Generalizability | 21 | Discuss the generalizability (external validity) of the study results. |  | 20 |
| **Other information** |  |  |  |  |
| Funding | 22 | Give the source of funding and the role of the funders for the present study and, if applicable, for the original study on which the present article is based. |  | 21 |
| *Ethics* |  |  | **nut-22.1** Describe the procedure for consent and study approval from ethics committee(s). | 23 |
| *Supplementary material* |  |  | **nut-22.2** Provide data collection tools and data as online material or explain how they can be accessed. | 23 |

**Supplementary Figure 1**


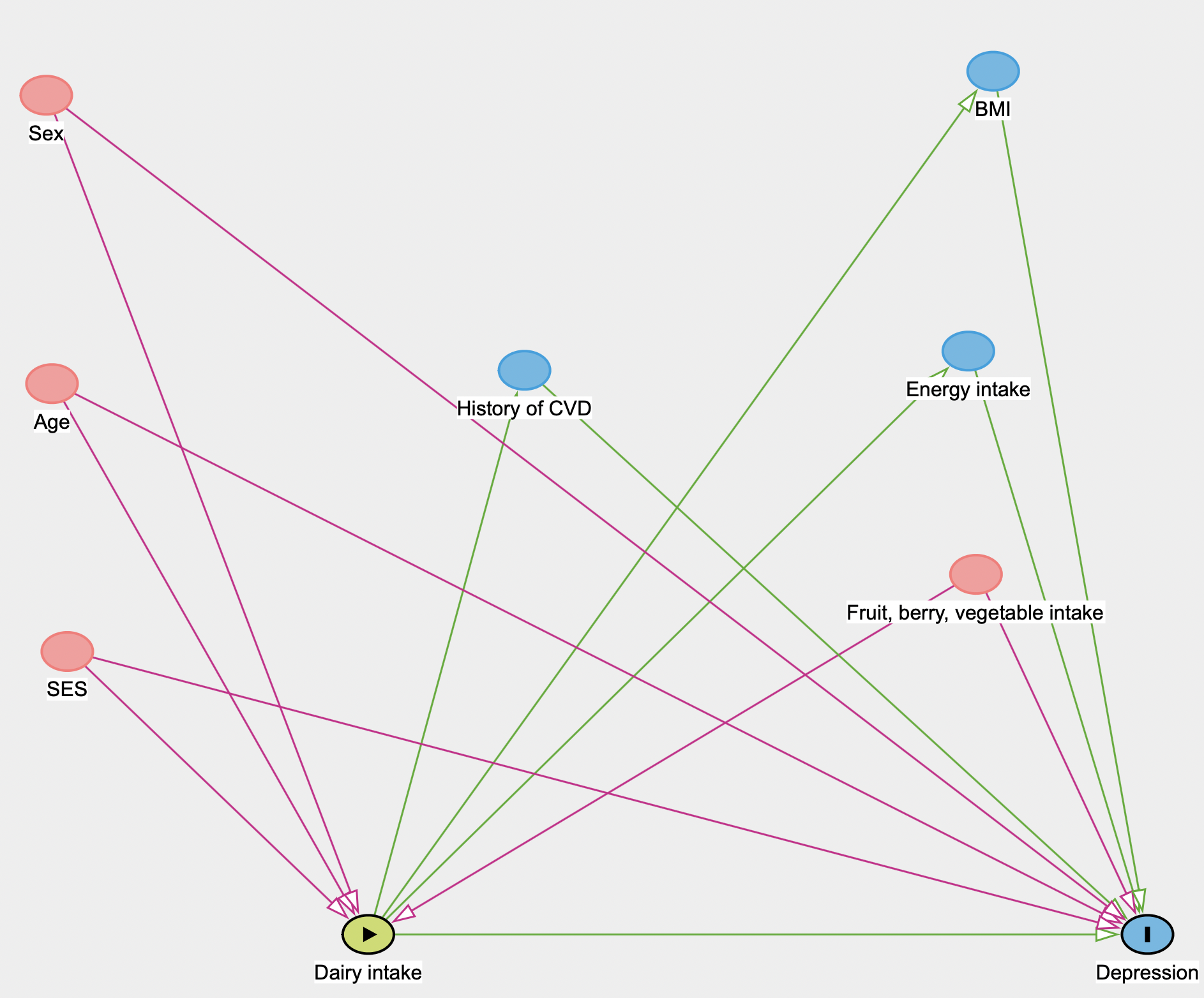


Adjustment (direct effect): Minimal sufficient adjustment sets for estimating the direct effect of Dairy intake on Depression:

- Age, BMI, Energy intake, Fruit, berry, vegetable intake, History of CVD, SES, Sex
